# A gene-rich, transcriptionally active environment and the pre-deposition of repressive marks are predictive of susceptibility to KRAB/KAP1-mediated silencing

**DOI:** 10.1186/1471-2164-12-378

**Published:** 2011-07-26

**Authors:** Sylvain Meylan, Anna C Groner, Giovanna Ambrosini, Nirav Malani, Simon Quenneville, Nadine Zangger, Adamandia Kapopoulou, Annamaria Kauzlaric, Jacques Rougemont, Angela Ciuffi, Frederic D Bushman, Philipp Bucher, Didier Trono

**Affiliations:** 1 School of Life Sciences, Ecole Polytechnique Fédérale de Lausanne (EPFL), Lausanne, Switzerland; 2 Frontiers-in-Genetics National Center of Competence in Research, Ecole Polytechnique Fédérale de Lausanne (EPFL), Lausanne, Switzerland; 3 Swiss Institute of Bioinformatics (SIB), Lausanne, Switzerland; 4 Department of Microbiology, School of Medicine, University of Pennsylvania, Philadelphia, PA, USA; 5 Institut de Microbiologie, Centre Hospitalier Universitaire Vaudois (CHUV), Lausanne, Switzerland

**Keywords:** KAP1, KRAB-zinc finger proteins, transcriptional repression, chromatin, heterochromatin, histone modifications

## Abstract

**Background:**

KRAB-ZFPs (Krüppel-associated box domain-zinc finger proteins) are vertebrate-restricted transcriptional repressors encoded in the hundreds by the mouse and human genomes. They act via an essential cofactor, KAP1, which recruits effectors responsible for the formation of facultative heterochromatin. We have recently shown that KRAB/KAP1 can mediate long-range transcriptional repression through heterochromatin spreading, but also demonstrated that this process is at times countered by endogenous influences.

**Method:**

To investigate this issue further we used an ectopic KRAB-based repressor. This system allowed us to tether KRAB/KAP1 to hundreds of euchromatic sites within genes, and to record its impact on gene expression. We then correlated this KRAB/KAP1-mediated transcriptional effect to pre-existing genomic and chromatin structures to identify specific characteristics making a gene susceptible to repression.

**Results:**

We found that genes that were susceptible to KRAB/KAP1-mediated silencing carried higher levels of repressive histone marks both at the promoter and over the transcribed region than genes that were insensitive. In parallel, we found a high enrichment in euchromatic marks within both the close and more distant environment of these genes.

**Conclusion:**

Together, these data indicate that high levels of gene activity in the genomic environment and the pre-deposition of repressive histone marks within a gene increase its susceptibility to KRAB/KAP1-mediated repression.

## Background

Gene expression is modulated through the alteration of chromatin states by epigenetic regulators. Krüppel-associated box zinc finger proteins (KRAB-ZFPs), which together constitute the single largest group of transcriptional repressors encoded by the human genome, partake in this process [1-3]. The KRAB-ZFP family is evolutionary recent and has expanded and diverged through multiple rounds of gene and segment duplications, to give rise to more than three hundred and fifty annotated members in humans [4-7]. Despite their abundance, KRAB-ZFPs and their transcriptional targets remain largely uncharacterized except for a few [8-10]. KRAB-ZFPs carry a C-terminal array of two to forty C2H2 zinc finger motifs, each potentially capable of recognizing a triplet of nucleotides in a sequence-specific manner [1], while their N-terminal KRAB domain recruits the KAP1 (KRAB associated protein 1) corepressor [11-14]. KAP1 (also named TIF1β, KRIP-1 or TRIM28) binds KRAB and homotrimerizes through its N-terminal RBCC (Ring finger/B box/Coiled-Coil) domain, while its C-terminus acts as a scaffold for various heterochromatin-inducing factors, such as heterochromatin protein 1 (HP1), the histone methyltransferase ESET (also known as SetDB1), the nucleosome-remodeling and histone deacetylation (NuRD) complex, the nuclear receptor corepressor complex 1 (N-CoR1) and, at least during early embryonic development, *de novo *DNA methyltransferases [15-22]. This results in local loss of histone acetylation, enrichment in histone 3 lysine 9 trimethylation (H3K9me3) and increased chromatin compaction [23,24].

Using chromatin immunoprecipitation (ChIP) and a tiling array, KAP1 has been documented to bind more than 7000 sites in a human testicular embryonal carcinoma cell line [25]. A more recent publication additionally revealed that KAP1 chromatin targeting falls into different categories, only a subset of which is dependent on its RBCC domain and consequently on its association with KRAB-ZFPs [26]. KAP1 is dynamically associated with both heterochromatin and euchromatin. It is thought to organize constitutive heterochromatin and to stimulate its propagation, as evidenced by its co-localization with HP1 in pericentromeric heterochromatin domains [16,27]. Using a combination of gene trapping and a drug-controllable KRAB-containing repressor, we recently demonstrated that KRAB/KAP1 can induce long-range repression through HP1-dependent heterochromatin spreading [28]. However, while some promoters located tens of kilobases (kb) from KAP1 docking sites were silenced by this mechanism, others were resistant. Here, we investigated the basis for this differential behavior by comparing the genomic context and the pre-existing levels of specific chromatin marks at repressed and non-repressed genes. This analysis revealed that genes most susceptible to KRAB/KAP1-induced silencing were in genomic regions of high gene activity. More specifically, repression was most efficient at sites with increased levels of pre-existing repressive histone marks at promoters and gene bodies, embedded within gene-rich regions with high levels of transcription.

## Results

### Characterization of thousands of KRAB/KAP1-targeted gene traps

**Figure 1:**
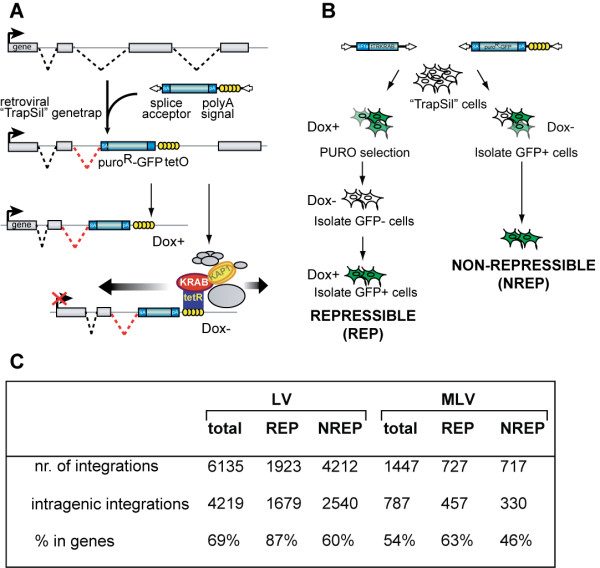
**Isolation of thousands of KRAB/KAP1 recruitment sites in genes with variable silencing phenotypes**. (A) Experimental setup used to target tTRKRAB to endogenous genes through the retroviral vector-based promoter trapping/silencing (TrapSil) system. *TetO*-containing gene traps, which carry the promoterless GFP-puro^R ^fusion reporter, are only expressed if they trap an actively transcribed gene. In the absence of doxycycline (Dox-), binding of the ectopic tTRKRAB repressor to *TetO *mediates silencing by recruiting KAP1 and associated heterochromatin-inducing factors. (B) Method used for isolating TrapSil HeLa cell subpopulations based on the effect of tTRKRAB binding on trapped gene expression. Repressible (REP) clones exhibit silencing of the reporter gene upon tTRKRAB binding, whereas reporter transcription of non-repressible clones (NREP) remains unaltered in this condition. (C) This table shows the outcome of the proviral integrant mapping and their distribution relative to genes. Mapping to the genome was performed with FetchGWI. The determination of intragenic integration sites was based on UCSC known genes. LVtotal and MLVtotal encompass all LV- and MLV -integrants. In addition, LV- and MLV- REP and NREP describe the repressible and non-repressible subsets of each vector type.

To study the impact of specific genomic features on KRAB/KAP1-induced silencing, we used the recently described trapping/silencing (TrapSil) system [28]. Here, retrovirally-trapped cellular promoters are exposed to a drug-regulated KRAB-containing repressor. The tTRKRAB protein contains the KRAB domain of the human KOX1 ZFP fused to the *E. coli *tetracycline repressor (tTR), and binds to Tet operator sequences (*TetO*) in a doxycycline (Dox)-controlled manner [29,30] (Figure 1A). We engineered retroviral-based gene trap vectors carrying tandem *TetO *repeats and a promoterless GFP-puromycin resistance fusion reporter. This design predicts that i) reporter expression occurs from the promoters of active genes targeted by the integrants ("trapping"), and ii) Dox withdrawal results in tTRKRAB binding to the *TetO *sites present in the provirus, thus exposing the trapped promoters to KRAB/KAP1-mediated silencing ("silencing") (Figure 1A). Using this experimental setup, we previously observed that while KRAB/KAP1 can act over long distances it is generally more effective when bound 20 kb or less from a promoter [28]. To study which other parameters might affect KRAB/KAP1-induced repression, we infected tTRKRAB-expressing HeLa cells with low doses of retroviral-based TrapSil vectors to ensure that only one integrant per cell was present. We made use of a combination of murine leukemia viral (MLV)- and lentiviral (LV)- based TrapSil vectors to obtain a greater diversity of targeted genes, since MLV tends to integrate close to active transcriptional start sites (TSS), while LV hits genes further downstream in their transcribed region [31,32].

Since we were interested in elucidating differences between KRAB/KAP1 repressible and non-repressible promoters and genes, we reasoned that "all or none" phenotypes would facilitate subsequent analyses. Therefore, we selected cells in which trapped promoters were highly active at baseline, and either strongly repressed ("repressed clones" containing a "repressing integrant") or almost completely resistant to this process ("non-repressed clones" containing a "non-repressing integrant") when the trans-repressor was allowed to bind its target (Figure 1B). More specifically, we isolated trapped integrants from a population of cells by puromycin selection in the presence of Dox, which impairs tTRKRAB binding and silencing. Then trapped integrants were subjected to subsequent rounds of cell sorting to isolate cells harboring gene traps with repressible promoters and reporter genes. These rounds first included the isolation of GFP negative cells when tTRKRAB was allowed to bind (Dox-), followed by the sorting out of GFP positive cells when its recruitment was inhibited (Dox+) (Figure 1B). Isolation of non-repressible genes was achieved by a similar approach. However, trapped cell populations were cultured in the presence of tTRKRAB binding (Dox-) and GFP positive cells, which did not silence reporter expression, were directly isolated after TrapSil vector infections (Figure 1B).

After the isolation of cell populations with differential silencing phenotypes, we mapped proviral integration sites, in order to identify the trapped genes. For this, we combined linker-mediated PCR (LM-PCR) of proviral-genomic junctions with massive parallel DNA pyrosequencing [31,33,34]. The amplified sites were mapped to the human genome with the FetchGWI software [35], and the UCSC known gene annotation was used to subsequently identify the trapped promoters (Figure 1C). We previously described that about 1 in 15 promoters trapped by MLV-TrapSil vectors were non-repressed by tTRKRAB, compared with approximately 1 in 5 for those captured by LV-based vectors [28]. Therefore, we isolated over 7000 integration sites, with an intentional bias for non-repressed clones to obtain integrant numbers comparable to their repressible counterparts. 69% of the promoter-trapping LV integrants mapped within annotated genes, whereas only 54% of their MLV counterparts did (Figure 1C, Additional File 1). This observation is in agreement with previous data indicating that parental MLV as well as MLV-based gene traps integrate in promoter proximal regions, which are less well annotated than gene bodies, which in turn are the preferential integration sites of LV and LV-based traps [36,37]. Consistently, we mapped 6135 LV-TrapSil integrants to the genome, 4219 of which were located within genes. In contrast, we only found 787 intragenic MLV-TrapSil integrants.

Prior to further analysis, we validated our experimental approach by deriving clones from each population. All of the 32 clones analyzed exhibited the expected silencing profile in flow cytometry measurements. Moreover, the clones comprised 10 non-repressed (LI I-X) and 8 repressed (LR I-VIII) LV-TrapSil clones, in addition to 8 non-repressed (MI I-VIII) and 6 repressed (MR I-VI) MLV-TrapSil clones, (Additional File 2). We also used ChIP analysis to verify that non-repressed genes properly recruited KAP1 and downstream effectors to their tTRKRAB docking site, in a doxycycline-dependent manner (Additional File 3). After this validation, we continued with the characterization of the genomic context of our KRAB/KAP1 repressible or non-repressible genes to find patterns correlating with silencing efficiency.

### Genomic environment of repressing and non-repressing gene trap integrants

We characterized the genomic environment of the integrants segregated according to their phenotype by using ROC (Receiver Operator Characteristic) curve analysis [38]. This type of analysis was previously used to identify the genomic features enriched around retroviral integration sites. This study confirmed that both MLV and LV preferentially integrate within transcriptionally active regions, and that this effect is augmented when integrants enabling reporter expression are selected [38]. In addition, this analysis also revealed that the effects of different genomic features on integration can change depending on the size of genomic segments in question [38]. Therefore, we included genomic intervals ranging from 0.1 kb to 10 Mb in our analyses.

**Figure 2:**
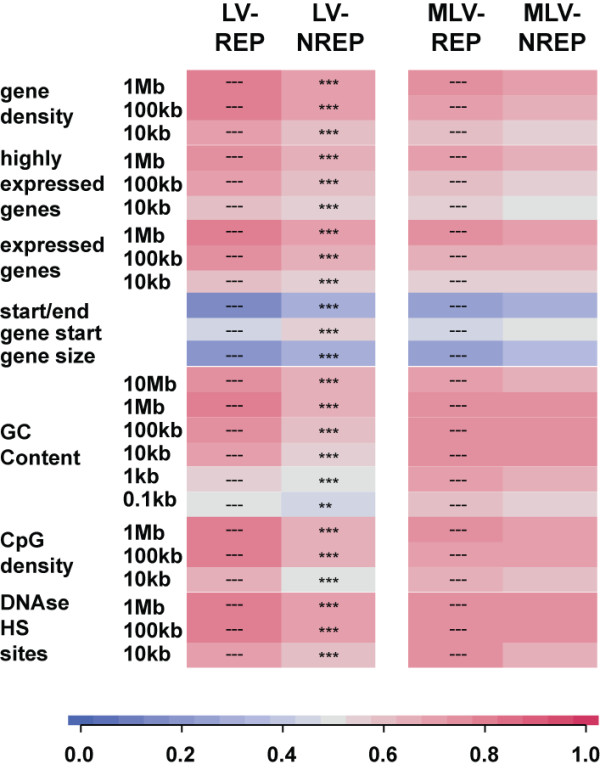
**Characterization of the genomic environment of KRAB/KAP1-docking integrants**. LV and MLV TrapSil integrants were split in repressing (REP) and non-repressing (NREP) groups according to the effect of KRAB/KAP1 recruitment on the trapped promoters. The genomic environment of the different proviral integrant groups was analyzed for the indicated genomic features by ROC curve analysis. This method serves to calculate the relative abundance of a given genomic feature around the integrants of a group for specific intervals. The resulting values are depicted in color-coded heat maps. 1 indicates that the specific feature is enriched in integrants, 0 means that it is depleted. Relative abundance scores of repressing and non-repressing integrants were compared for each trapping vector subtype and the statistical method used included the non-central chi-square test (** p < 0.01; *** p < 0.001). The different genomic feature categories tested were: "gene density", with all of the Refseq annotated genes; the "highly expressed genes" and "expressed genes" group, including genes expressed in the top 1/16^th^, or the top 1/2 of all genes measured in a transcriptional profiling analysis; *"*start/end", including the distance to the nearest transcriptional start (TSS) or stop site; "gene start", including the distance to the nearest TSS; "gene size" was the average size of the targeted genes and was only analyzed for intragenic integrants; "GC content", included the density of GC nucleotides, which are more abundant in gene-rich regions; "CpG density", contained the frequency of CpG dinucleotides, mostly present at promoters; "DNAse HS sites", included the number of DNAse I hypersensitive sites, frequently associated with gene regulatory regions.

In order to characterize the genomic features surrounding the integrants in our different TrapSil groups, we made use of the same approach. We first calculated the area under the ROC curve, which is a common measure of a predictor variable's ability to discriminate between two classes of events. In our case we compared the average enrichment of a given feature at a set of genomic sites (such as integration sites in our case) relative to that of a set of random matched control sites. The read-out of this comparison is illustrated in color-coded heatmaps, where each rectangle represents the specific enrichment of a feature within the indicated intervals of distance. The relative enrichment between the integration and control site group is scored on a scale from 0 to 1. 1 is scored when a specific feature is enriched in the experimental integrants when compared to matched controls, 0 is scored when the opposite is true. A value of 0.5 indicates no difference between the two groups. The patterns of the genomic features surrounding the TrapSil integrant groups largely reflected the preferential genomic environment associated with either LV or MLV integrations (for values see Additional File 4). It included a preference for both retroviruses for active genes, in addition to their differential targeting to gene bodies and promoters, respectively. This is reflected by an increased enrichment of CpG islands and DNase I sites at short intervals around MLV integrants when compared to LV integrants. This difference is lost when larger intervals are included in the ROC area calculation (Figure 2).

We then compared the ROC values, which are proportional to the levels of genomic features at these sites, between TrapSil groups harboring differential susceptibilities to KRAB/KAP1-silencing. We did this by making relative comparisons between a chosen reference and other gene groups. The reference gene groups are indicated by the symbol "---" within the whole results section. Using this approach we compared the levels of specific genomic features between the respective REP gene group and their corresponding NREP counterpart. When statistical differences were assessed, we found that LV-TrapSil repressing integrants were located within gene-denser genomic regions than non-repressible integrants (Figure 2). Furthermore, the environment of repressing LV integrants was enriched in CpG and DNase I sites, as well as in highly expressed genes (based on publicly available microarray data), compared to that of non-repressing LV integrants. While all the described parameters were statistically significantly different between repressible and non-repressible LV traps, comparisons of their MLV-TrapSil counterparts did not reach significant differences, although it showed similar trends (Figure 2). Therefore, a positive correlation between gene activity in the environment of the targeted transcriptional unit and efficient KRAB/KAP1-mediated silencing is established. The lack of significance between the MLV repressible and non-repressible TrapSil groups could be due to smaller integrant numbers or could reflect the presence of other uncharacterized features affecting KRAB/KAP1 recruitment, including the on average closer proximity of MLV integrants to promoters.

### Genomic features of matched repressed and non-repressed transcriptional units

**Figure 3:**
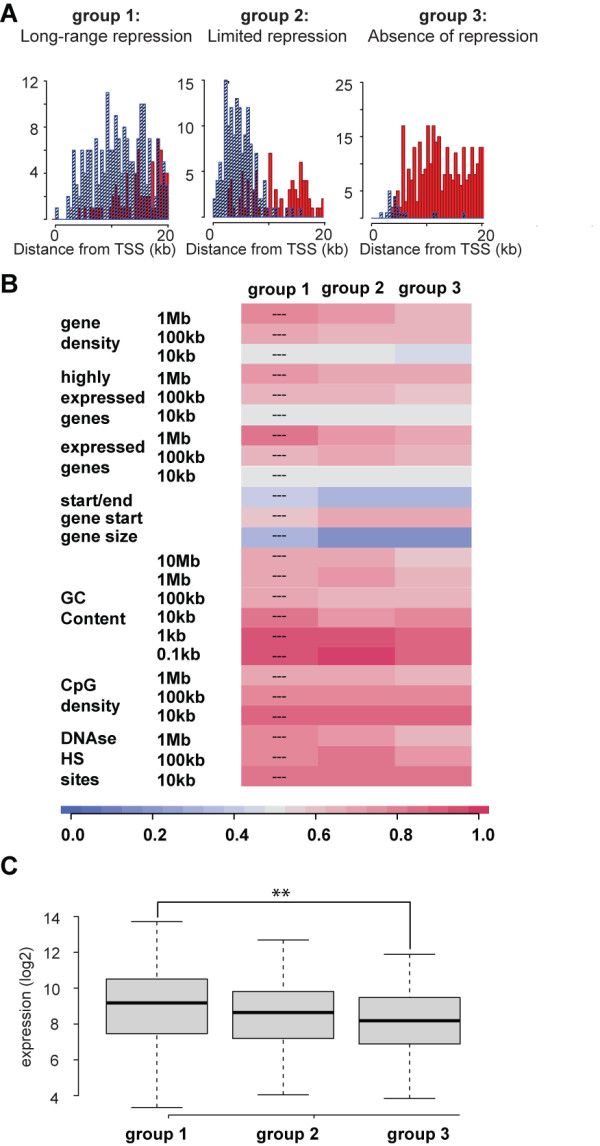
**Generation and characterization of matched gene groups displaying differential KRAB/KAP1 silencing phenotypes**. (A) Matched gene groups with differential KRAB/KAP1-silencing phenotypes. The cumulative histograms illustrate the distribution of repressible (blue) and non-repressible (red) LV-TrapSil and MLV-TrapSil integrants in the transcribed region of genes trapped multiple times. Three groups were distinguished based on the pattern of repressible and non-repressible integrants over 20 kb. Group 1 genes harbored mainly repressing integrants ("long-range repression"), while group 3 contained mostly non-repressible integrants ("absence of repression"). Group 2 genes exhibited an intermediate phenotype, with repressing integrants clustered over the first 10 kb of their transcribed region ("limited repression"). (B) The three gene groups (group 1: "long-range repression", group 2: "limited repression" and group 3: "absence of repression") were analyzed by ROC curves as described in Figure 2 for genomic features over various DNA stretches. (C) Comparison of mean expression levels of the three gene groups based on publicly available microarray data. Group 1: "long-range repression", group 2: "limited repression" and group 3: "absence of repression". Statistical comparisons were made with a non-parametric Wilcoxon test. P-Value Legend: ** p < 0.01.

Repressing integrants were on average closer to the transcriptional start site of their targets, compared to non-repressible integrants (Figure 2). This finding is consistent with results from our previous analyses, which revealed that KRAB/KAP1-induced repression was more likely if gene traps were located closer to trapped promoters [28]. Therefore, the described integrant-centered analysis may suffer from potential biases linked to this spatial factor. We thus repeated our analyses focusing on genes that had a minimal size of 20 kb, a single known TSS, and were frequently targeted by our trapping vectors, that is, hit at least three times in our series. We then classified these genes into three subgroups according to their susceptibility to KRAB/KAP1-induced silencing expressed as a function of the distance between KRAB-docking integrant and trapped promoter. This led to the identification of 70 genes that supported long-range repression, that is, for which most integrants located within 20 kb of the TSS were repressing (group 1); 77 genes supporting limited range repression, with silencing occurring mainly when integrants were located 10 kb or less from the TSS (group 2); and 80 genes resistant to repression, where no significant silencing occurred irrespective of the distance between the TSS and the KRAB/KAP1-docking sites (group 3) (Figure 3A). Of note, there was no difference between the expression patterns of these genes in different tissues, indicating that these gene groups did not differ in being essential or not for cellular maintenance (data not shown).

The genomic context of the three gene groups was reminiscent of observations made in the integrant-centered analysis (Figure 2), with genes from group 1 being in gene-richer and transcriptionally more active environments, and surrounded by a higher density of DNase I hypersensitivity sites (Figure 3B). These associations, however, did not reach statistical significance. Importantly, no difference in distance between repressor binding site and the trapped promoter was apparent when comparing the three groups, eliminating concerns about this potential bias for subsequent analyses of these genes (Figure 3B). When we examined the expression levels of the different gene groups, we found that genes supporting long-range repression (group 1) were on average more highly expressed than genes that did not enable KRAB/KAP1-mediated repression (group 3) (Figure 3C). Therefore, KRAB/KAP1-mediated silencing seems to be more effective in regions of high gene activity. To further consolidate this result, we assessed the levels of different chromatin features, correlating with transcriptional activation or repression in our different gene groups.

### Chromatin features of matched repressed and non-repressed transcriptional units

We first assessed the levels of putative barrier elements such as CTCF, H3.3/H2Az or chromatin modifiers in the different groups [39-41]. This was achieved by utilizing published datasets, which were used to calculate the relative abundance of these features by ROC curve analysis and by comparing these values between the groups. There was no differential association with either one of the three gene groups for the intervals tested (Additional File 5).

**Figure 4:**
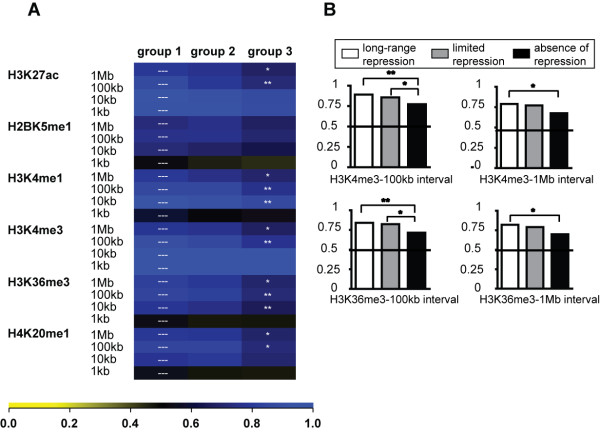
**Characterization of the chromatin environments of the matched gene groups with respect to active histone marks**. (A) The levels of specific posttranslational histone modifications around the promoters of the different gene groups were calculated over different DNA intervals ranging from 1 kb to 1 Mb. The gene groups included group 1: "long-range repression", group 2: "limited repression" and group 3: "absence of repression" and ROC curve analyses was employed. Briefly, the illustrated heat maps, contain squares giving the relative abundance of the studied histone mark. 1 is scored when the modification is enriched in the gene group when compared to a control group, while 0 indicates depletion of the mark. 0.5 is scored when there is no difference. Non-central chi-square statistical analysis compared differences between the repressible group 1 and the non-repressible group 3. P-Value Legend: * p < 0.05; ** p < 0.01. The histone modifications in the analysis included H3K27ac, H2BK5me1, H3K4me1, H3K4me3, H3K36me3, H4K20me1, which are mostly found within active chromatin. (B) Relevant histograms representing the relative H3K4me3 and H3K36me3 values over 100 kb and 1 Mb, which reached statistically relevant differences when all three gene groups were compared.

We then assessed the abundance of a series of histone modifications present at an interval of 1, 10 or 100 kb or 1 Mb around our promoters of interest. We first measured the levels of posttranslational histone modifications correlating with active gene expression, including histone H3 lysine 27 acetylation (H3K27ac), H2BK5 monomethylation (H2BK5me1), H3K4 mono- and trimethylation (H3K4me1, me3), H3K36me3 and H4K20me1. For this we generated genome-wide histone modification maps using a ChIP coupled to deep sequencing (ChIPseq) approach in HeLa cells. Then we used ROC curve-based heatmaps to obtain relative enrichment values for the three groups. When the long-range repressing group 1 was compared to the non-repressing group 3 most of the active histone modifications were enriched in group 1 (Figure 4). This was the case for smaller (10 kb) and larger intervals (100 kb, 1 Mb), consistent with the idea that KRAB/KAP1-repressible genes reside in regions of very active chromatin both on a local and a more global scale (Figure 4A).

**Figure 5:**
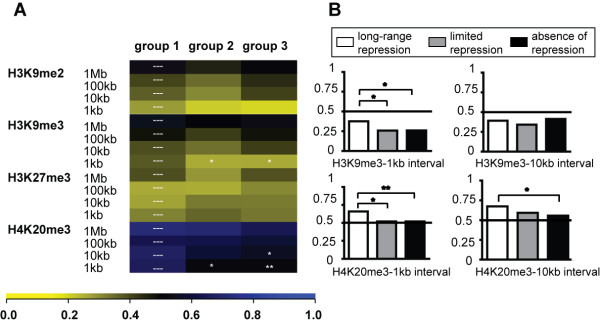
**Characterization of the chromatin environments of the matched gene groups with respect to silent histone marks**. (A) The levels of histone marks associated with silent chromatin were analyzed at specific intervals around the promoters of the three gene groups (group 1: "long-range repression", group 2: "limited repression" and group 3: "absence of repression"). The calculations were based on ROC curve analysis as described in Figure 4. Non-central chi-square statistical analysis compared differences between group 1 and group 2, and group 1 and group 3. The p-Value Legend is * p < 0.05; ** p < 0.01. The histone modifications in the analysis included H3K9me2, H3K9me3, H3K27me3 and H4K20me3, mainly associated with silent chromatin. (B) Relevant histograms representing relative H3K9me3 and H4K20me3 values over 1 kb and 10 kb, which reached statistically relevant differences when the three groups were compared.

We then measured the levels of histone modifications normally present at silent genes, such as H3K9me2/me3, H3K27me3 and H4K20me3. For this we generated histone modification maps by ChIPseq in HeLa cells or relied on a published dataset for the distribution of H3K27me3 in this cell line [39]. Furthermore, group 1 genes were comparatively less depleted in H3K9me3 at the TSS and enriched in H4K20me3 at the promoter and within a 10 kb distance from the TSS, compared with genes from groups 2 and 3 (Figure 5). Therefore, promoters sensitive to KRAB/KAP1-mediated repression harbor increased levels of some silent histone marks, which are embedded within a domain of very high gene activity.

## Discussion

Previous analyses on the mechanisms of KRAB/KAP1-mediated gene regulation have mostly examined the impact of this system on the expression of transfected promoter-reporter units. Here, we investigated KRAB/KAP1-induced changes within the context of endogenous genes. Using a combination of promoter trapping and drug-controllable KRAB/KAP1 recruitment, we previously observed that this complex, when docked to the bodies of transcriptionally active genes, could induce silencing over distances of several tens of kilobases [28]. However, we had also noted that repression was more efficient if the distance between the effector and the promoter was less than 20 kb. Furthermore, a significant fraction of trapped promoters/KRAB docking loci escaped these rules, suggesting other counteracting influences. The present large-scale comparison of the genomic features of KRAB/KAP1-responsive and KRAB/KAP1-resistant transcriptional units identified by our gene trap system reveals a positive correlation between efficient KRAB/KAP1-mediated repression of trapped promoters and i) a gene-richer and transcriptionally more active genomic context, ii) a more euchromatic environment, and iii) the pre-existence of some repressive marks at and around the promoter.

Comparing KRAB/KAP1-repressed and non-repressed genes gave no indication for a role of putative obstacles to the spread of heterochromatin, such as CTCF binding, accumulation of H3.3/H2Az or recruitment of HATs (reviewed in [42,43]). This is consistent with the observation that CTCF recruitment to the HS4 region of the chicken β-globin locus can be prevented without abrogating the barrier function of this DNA sequence [44]. However, it is at odds with a recent study presenting CTCF as a marker of transition between euchromatic and heterochromatic regions [39]. A model reconciling these findings would be that CTCF acts as an H3K27me3 heterochromatin-specific barrier yet has no effect on H3K9me3-based heterochromatin propagation. However, it should be emphasized that our analysis was limited to the transcribed region of genes owing to our gene trap-based approach, precluding overly general conclusions on the possible role of barrier elements.

Although both repressed and non-repressed genes were situated within euchromatic regions, as expected from the promoter-trapping approach used for their selection, we observed significant differences in both their local and broader chromatin environments. Repressed genes were in regions containing generally higher levels of major euchromatin-associated marks and higher levels of transcription compared with non-repressed genes. Therefore, there is a positive correlation between efficient KRAB/KAP1-silencing and high gene activity. This is suggestive of a model whereby genes situated in more heterochromatic environments can only be highly expressed if endowed with an intrinsic ability to resist repressive influences, while genes located in more euchromatic environments do not need such protective mechanisms [45]. Consistently, in our analysis KRAB/KAP1-resistant units were on average closer to telomeres than their KRAB/KAP1-susceptible counterparts, although this difference did not reach statistical significance (data not shown).

Genes repressed by the TrapSil system also carried higher levels of the repressive marks H4K20me3 and H9K9me3 at baseline at and around their promoters, compared with their repression-resistant counterparts. Noteworthy, these contrasting chromatin configurations were not only observed when comparing a selected set of multiply hit repressed and non-repressed genes (Figure 5), but were also present in the complete pools of repressing and non-repressing integrants (Additional File 6). Interestingly, a recent analysis of the chromatin structure of zinc finger genes found that high levels of both H3K36me3 and H3K9me3 co-localized at the 3' exons of these genes [46]. Since KRAB-ZFP genes, which belong to this gene family, are endogenous targets of KRAB/KAP1-repression [25,47], we performed the same analysis in our HeLa cell system and reproduced the same result (Additional Files 7 and 8). Therefore, the high levels of both H3K9me3 and H3K36me3 at KRAB-ZFP gene bodies may be necessary for efficient KRAB/KAP1-induced heterochromatin spreading. The finding that the repressive H3K9me3 and the activating H3K36me3 marks are not co-regulated further supports this hypothesis [46], since high levels of H3K36me3, which positively correlate with active transcription, may independently enhance the spread of H3K9me3 at KRAB-ZFP genes. This model is reminiscent of results obtained from the TrapSil analysis, where high levels of both active and repressive histone marks can be seen in genes that accommodate KRAB/KAP1-mediated heterochromatin spreading and silencing.

A difference between genes targeted by our TrapSil system and endogenous KRAB-ZFP genes lays in the finding that the latter do not seem susceptible to KRAB/KAP1-mediated long-range repression [28,46,47]. This may be due to the use of our ectopic repressor system. Alternatively, certain endogenous promoters may be resistant to KRAB/KAP1-induced heterochromatin spreading. A possible factor in this process is the H3K9me1/2 demethylase PHF8 [48]. Active H3K9 demethylation may prevent the heterochromatization of KRAB-ZFP promoters and subsequent transcriptional silencing. This idea is consistent with recent PHF8 genome-wide binding data that showed it locating to the promoter regions of zinc finger-encoding genes [49].

Other mechanisms potentially involved in conferring resistance to KRAB/KAP1-mediated silencing are suggested by the analysis of genes that were hotspots of proviral TrapSil targeting and carried both repressible and non-repressible integrants (Additional File 9). In this subgroup, the repressible integrants generally clustered closer to the promoter than their non-repressible counterparts, consistent with the overall observation that silencing is most efficient when KRAB/KAP1 is recruited in the proximity of the affected promoter. In some cases, however, the distributions of repressible and non-repressible integrants overlapped within the same gene. This could reflect the differential susceptibilities of the two alleles of a gene to KRAB/KAP1-mediated repression, somewhat reminiscent of what is observed with imprinting, a process that involves a KRAB-ZFP [9,50]. Additionally, cells within a population may be heterogeneous for the chromatin status of specific loci, which in turn might impact on the consequences of KRAB/KAP1 recruitment. Such a phenomenon would be comparable to variegation, where particular genes are differentially expressed amongst cells of an otherwise apparently homogeneous population [51].

## Conclusions

In summary, the present work indicates that the impact of KRAB-mediated docking of KAP1 on the expression of targeted genes is more variable than previously suspected. It further reveals reciprocal influences between the functional outcome of KRAB/KAP1 recruitment to DNA and the chromatin features of the involved loci. More broadly, the approach described in the present study, which combined an analysis of the functional consequences of exogenously introduced *cis*-acting KRAB/KAP1-recruiting sequences with an examination of the transcriptional activity, genomic context and chromatin features of targeted loci, could be fruitfully applied to the study other epigenetic regulators.

## Methods

### Vectors

pLV-tTR-KRAB-Red was previously described [52]. pLtTR-KRAB-NG95 was cloned through ligation of a BamHI/XhoI digested MLV-based pNG95 [53] with a compatible tTR-KRAB amplicon with BamHI/XhoI sites added by PCR (primer sequences see Additional File 10). To construct LV- and MLV-based TrapSil vectors, published gene trap vectors [37] were modified by PCR-based mutagenesis (Stratagene mutagenesis kit). A BlpI restriction site was introduced into the MLV U3 region of 3'LTRs (MLV: BlpI Primers MLV Trap F/R - Additional File 10), whereas a SpeI site was introduced in the LV U3 region of 3'LTR (Primers HIV Trap F/R - Additional File 10), these new sites were then used to insert 7 repeats of TetO. LV- and MLV-based particles were produced and titered as described elsewhere http://tcf.epfl.ch/page-6764-en.html. The WPRE of LV-TRAPSIL, the GAG remnant of MLV-TRAPSIL, and the Albumin gene served for proviral and cellular genome quantification by Taqman.

### Cell culture and Fluorescence activated cell sorting (FACS)

HeLa cells were grown under standard conditions. Doxycycline (Sigma-Aldrich) was used at a concentration of 1 μg/mL. Clonal tTRKRAB-expressing HeLa cell lines dsRK4 (pLV-tTR-KRAB-Red, LV-backbone) and KiN1.25 (pLTetR-KRAB-NG95, MLV-backbone) were derived after infection with pLV-tTR-KRAB-Red or pTetR-KRAB-NG95, respectively. The LV based HeLa dsRK4 clone contains approx. 15 vector copies as titrated by Taqman and was used for MLV-TRAPSIL assays while the MLV-based KiN1.25 clone contains 10 vector copies and was used for all LV-TRAPSIL assays. In view of this mapping strategy, 2 × 10^8 ^dsRK4 or KiN1.25 HeLa cells were infected with 1.6 × 10^6 ^MLV-TrapSil or LV-TrapSil infectious particles, respectively, with a multiplicity of infection of 0.04. Cells were sorted based for GFP expression by using the Beckton Dickinson FACSVantage SE turbo Sorter with Diva Option. Flow Cytometry analyses were performed on BD FACScan flow cytometer.

### Quantitative PCR (qPCR)

qPCR reactions were carried out with a standard PCR program in ABI PRISM 7900 HT in duplicates or triplicate using either SYBR green detection 1× Power Sybr or 1× Taqman Universal Mix, No AmpErase (Applied Biosystems). Primers were used at a final concentration of 100 nM. When SYBR analysis was performed, cycling reactions were followed by a dissociation curve analysis to validate specificity of amplified products. The increase in fluorescence was analyzed with the SDS software, version 2.2.2 (Applied Biosystems). For all amplification plots the baseline data were set with the automatic cycle threshold function. Primer sequences for all qPCR reactions are listed in Additional File 10.

### Linker-mediated PCR (LM-PCR), 454 pyrosequencing and data processing

LM-PCR was used to map integration sites following a previously described protocol [31,33,34]. Briefly, 10 μg of genomic DNA (DNeasy, Qiagen) was digested with MseI. Fragments were ligated to a linker and were digested with DpnI and SacI (LV-TrapSil) or SpeI (MLV-TrapSil) to avoid contaminations with bacterial plasmids and to avoid cloning of internal vector fragments. Nested PCR then served to amplify TrapSil vector-gDNA junctions (Takara Advantage 2 kit). Amplicons ranging between 100 and 400 bp were purified, quantified and sent for pyrosequencing at GATC biotech (Konstanz, Germany). Raw sequences were downloaded from the GATC biotech website and converted to FASTA files. Sequences having exact pyrosequencing reaction primers (F: primer A; R: primer B, Additional File 10) were selected and others discarded. Selected sequences were then categorized according to barcode for TrapSil vector type and integrant type (barcodes: LI: TGAC/AGTC; LR: CTGA; MI: TCGA/AGCT; MR: GTAC). After classification, all primer sequences and viral vector overhangs were trimmed yielding only genomic DNA sequence. The 20 bases adjacent to primer B before trimming were used as tags for mapping the inserts to the human genome assembly hg18. The mapping was done using FetchGWI tolerating at most 2 mismatches [35].

Integration site mapping in genes: Integrant orientation was annotated as determined during sequence processing. UCSC known gene [54] were downloaded from UCSC tables with transcript start (Tsx), transcript end (Tsend) and gene orientation. Only integrants mapping with correct orientation within a gene were mapped relative to it. In a second step, a non-redundant gene list was generated (from the original UCSC Gene list) using an aggressive clustering strategy, which groups all transcripts that directly or indirectly (through other transcripts) overlap on the same strand of the same chromosome. In the non-redundant gene list we recorded the 5'most Tsx position and the 3'most Tsend position for each cluster. For analysis of the integrant distance to gene promoters, we considered only integrants falling within the transcribed region of the same gene. Files containing integration sites (sequence-mapping from Insipid, LV_LUI (LV irrepressible), LV_LUR (LV repressible), MLV_MUI (MLV irrepressible), MLV_MUR (MLV_repressible)) and gene groups (20 KB promoter classes with at least 3 integration sites: group 1: "long-range repressible), group 2: "short-range repressible", group 3: "long-range irrepressible") can be found under http://ccg.vital-it.ch/KAP1/.

### Receiver Operator Characteristic (ROC) curve analysis

Data analysis was based on a "nested case control" strategy using a collection of TSS characterized by a given behavior with respect to repression along with control sites sampled from the genome to make inferences about the probability of a TSS to display a given response to repression based on genomic/epigenetic features characterizing its environment. More detailed description of statistical basis for this analysis can be found in [38]. Data were analyzed using the R language and environment for statistical computing/graphics version 2.3.0 and several contributed packages. Empirical ROC curve areas were calculated for datasets that used random genomic controls, in which case each TSS of a cluster was compared only with its matched controls to determine the proportions of controls whose values equaled or exceeded that of TSS [55]. Annotations of genomic features were obtained as described previously [38]; the chromatin features analyzed came from ChIP-seq data generated in this and other studies [39-41].

### Chromatin immunoprecipitation (ChIP) and ChIP followed by sequencing (ChIP-Seq)

ChIP reactions were performed according to published protocols with minor modifications (http://www.millipore.com/userguides/tech1/mcproto407 and http://cshprotocols.cshlp.org/cgi/content/full/2009/6/pdb.prot5237), using antibodies listed in Additional File 11, either native or pre-bound to beads. For Histone modifications, 2 × 10^7 ^HeLa cells were trypsinized and resuspended in MNase buffer. 1 U MNase (Roche) was added for 10 min and adding EDTA to a final of 10 mM arrested the nuclease reaction. Chromatin was sonicated with a Branson digital sonicator (model 250) on ice three times for 20 s and then dialyzed against RIPA with AEBSF protease inhibitor 0.2 mM for 1 h. The chromatin was pelleted after dialysis; glycerol was added to the supernatant to a final 5% concentration and the chromatin was stored at -80°C. 500 ul was incubated with AB-specific pre-coated beads over night (IP). Complexes were washed, eluted, purified, precipitated and resuspended in 50 ul H2O. For KAP1 ChIPs, approximately 2 × 10^7 ^cells were cross-linked with 1% formaldehyde for 8 min at RT, quenched by adding glycine and rinsed with PBS, before shearing by sonication with a Branson digital sonicator (model 250) on ice four times for 20 s at 30% intensity. 100 μl of sonicated chromatin was directly de-crosslinked and used as the total input (TI) reference in qPCR analysis at a dilution of 1:100. 100 μl of sonicated chromatin was used for each ChIP reaction and was diluted in 900 μl dilution buffer and precleared with 80 μl salmon-sperm DNA protein A agarose beads (Upstate). Chromatin-antibody complexes were captured washed and eluted with 100 mM NaHCO_3_, 1% SDS. Cross-links between DNA and proteins were reversed by addition of NaCl and incubation at 65°C. DNA was precipitated after incubation with RNase A (Sigma) and Proteinase K (Roche) and resuspended in 50 μl H_2_O and subjected to qPCR analysis. qPCR is described above and primers are listed in Additional File 10. Negative control reactions without antibody were run for each sample and in all cases gave negligible results. To validate the relative enrichment of proteins or specific histone modifications at a given sequence a ratio between the relative quantities of IP and TI was established.

The sequencing libraries from all ChIP products were prepared using the ChIP-seq Sample Preparation Kit (Illumina; San Diego, California, USA; Cat. No. IP-102-1001) according to the protocol supplied with the reagents and using 10 ng of ChIP sample quantified using the Qubit fluorometer (Invitrogen; Carlsbad, California, USA). One lane of each library was sequenced on the Illumina Genome Analyzer IIx using the Single-Read Cluster Generation Kit v2 (Cat. No. FC-103-2001) and 36 Cycle Sequencing Kits v3 and v4 (Cat. Nos. FC-104-3002 and FC-104-4002). Data were processed using the Illumina Pipeline Software v1.5.1. Illumina GAII data were mapped to the genome with Bowtie http://bowtie-bio.sourceforge.net/index.shtml. All output files were converted to processable file formats (SGA) for subsequent bioinformatics analysis described below. Enrichment of genomic and chromatin features was assessed with the ChIPcor web-based tool http://ccg.vital-it.ch/chipseq/chip_cor.html. All files were converted into SGA format, settings included: sort input: on, strand option: oriented for references files (gene clusters TSS and poly-A sites) and any for all target features, range was set at -40 kb to +40 kb, window size was at 500/50 (for graphic or statistical analysis) and cut-off value was 1. Raw and processed sga files can also be found under http://ccg.vital-it.ch/KAP1/ and were used as follows: "SGA files for Zhao-produced CTCF and H3K27me3 genome wide" include HeLa-CTCF.sga.gz, HeLa-H3K27me3.sga.gz data; "H3.3-H2A.Z double ChIP (Zhao et al)" contain GSM335958.sga.gz files; "SGA files from genome-wide mapping of HATs and HDACs in human CD4+ T cells" [41]: contain CD4-Tip60.sga.gz; "SGA files for histone modification profiles (ChIP-Seq data)": contains H3K27ac: H3K27acpf.sga, H2BK5me1: H2BK5me1pf.sga, H3K4me1: H3K4me1pf.sga, H3K4me3: H3K4me3p.sga, H3K36me3: H3K36me3b.sga, H4K20me1: H4K20me1b.sga, H3K9me2: H3K9me2p.sga, H3K9me3: H3K9me3pf.sga, H4K20me3: H4K20me3b.sga.

### Microarray data

HU133a arrays for HeLa cells were downloaded from GEO/NCBI [56,57] and data were extracted and normalized using RNA Robust Multichip Average (Quantile normalization) [58]. Specific gene expression scores were extracted and normalized average values for 4 different arrays were calculated. Statistical comparisons were done with the non-parametric Wilcoxon test.

## Competing interests

The authors declare that they have no competing interests.

## Authors' contributions

Conceived and designed the experiments: SM, ACG, DT. Performed the experiments and computational analyses: SM, ACG, GA, NM, NZ, AK, AK, JR. Contributed reagents/materials/analysis tools: SQ, AC, FDB, PB. Wrote the paper: SM, ACG, DT. All authors have read and approved the final manuscript.

## Supplementary Material

Additional file 1 **List of mapped integrants and integrant gene groups**. Click here for file

Additional file 2 **KRAB/KAP1 repression profiles from clones isolated from repressible and non-repressible TrapSil populations**. Reporter gene activity in the presence and absence of doxycycline (Dox) was monitored in clones derived from repressible and non-repressible TrapSil populations. Click here for file

Additional file 3 **KAP1 and H3K9me3 are present at non-repressible clones in the presence of repressor binding**. Chromatin-immunoprecipitation (ChIP) in combination with qPCR was used to verify proper KAP1 recruitment to gene traps in the absence of doxycycline in non-repressible clones. In addition, levels of H3K9me3 monitored the enzymatic activity of the KRAB/KAP1 silencing complex. The relative enrichment was calculated as a percentage of the total input (% of total input). Click here for file

Additional file 4 **Values from Receiver Operator Characteristic (ROC) curve analysis**. Click here for file

Additional file 5 **Heterochromatin barrier elements in the environment of the matched gene groups**. The relative level of barrier elements was assayed in the three gene groups (group 1: "long-range repression", group 2: "limited repression" and group 3: "absence of repression"). Calculations were based on ROC curves as described in Figure 3 and included various DNA stretches around the promoters of the different gene groups. Non-central chi square statistical analysis indicated no differences between the groups. The features considered in the analysis were (A) Histone variants associated with active remodeling (H3.3, H2Az), (B) CTCF or (C) chromatin modifiers, such as the histone acetyltransferases p300, TIP60, PCAF and MOF, in addition to the histone deacetylases HDAC1, 2, 3, and 6. The genome-wide binding data for these factors came from published work in HeLa cells (H3.3, H2Az, p300) or CD4 cells (TIP60, PCAF, MOF, HDAC1, 2, 3 and 6). Click here for file

Additional file 6 **Characterization of the chromatin environment of all KRAB/KAP1-docking integrants**. LV and MLV TrapSil integrants were split in repressing (REP) and non-repressing (NREP) groups according to the effect of KRAB/KAP1 recruitment on the trapped promoters. The chromatin environment of the different proviral integrant groups was analyzed for the indicated features by ROC curve analysis. (A) The histone modifications in the analysis included H3K27ac, H2BK5me1, H3K4me1, H3K4me3, H3K36me3, H4K20me1, which are mostly found within active chromatin. (B) The histone modifications in this analysis included H3K9me2, H3K9me3, H3K27me3 and H4K20me3, mainly associated with silent chromatin. Non-central chi-square statistical analysis compared differences between the integrant groups with the differential silencing phenotypes. P-Value Legend: * p < 0.05; ** p < 0.01; *** p < 0.001. Click here for file

Additional file 7 **Characterization of the chromatin state of KRAB-ZFP genes**. Illustration of H3K9me3 and H3K36me3 enrichments at KRAB-ZFP gene bodies in HeLa cells. KRAB-ZFP gene lengths were equalized by division into 40 bins and included a 2 kb flanking region on both sides of the genes. See Additional File 8 for the list of KRAB-ZFP genes, which were included in the analysis. Click here for file

Additional file 8 **List of known and putative KRAB-ZFP genes included in the analysis**. Click here for file

Additional file 9 **Genes with multiple TrapSil integrants of different silencing phenotypes**. Graphic representation of 6 genes, where multiple TrapSil integrations of different phenotypes have occurred: calnexin precursor (CANX), heterogeneous nuclear ribonucleoprotein A2/B1 (HNRNPA2B1), karyopherin beta 1 (KPNB1), nucleophosmin 1 (NPM1), Y-box binding protein 1 (YBX1), laminin alpha 5 (LAMA5). The integrants with a repressible phenotype are depicted as black lines above the baseline (y = 0), whereas the non-repressible counterparts are depicted as light grey lines below the baseline. Some intragenic sites carried multiple but distinct proviral integrants and subsequently harboured more than 1 integrant count (see y-axis). Click here for file

Additional file 10 **List of primers used in this study**. Click here for file

Additional file 11 **List of antibodies used in this study**. Click here for file

## References

[B1] Urrutia R KRAB-containing zinc-finger repressor proteins Genome Biol 2003 4 10 231 10.1186/gb-2003-4-10-231 14519192 PMC328446

[B2] Ding G Lorenz P Kreutzer M Li Y Thiesen HJ SysZNF: the C2H2 zinc finger gene database Nucleic Acids Res 2009 37 Database D267 273 10.1093/nar/gkn782 PMC2686507 18974185

[B3] Emerson RO Thomas JH Adaptive evolution in zinc finger transcription factors PLoS Genet 2009 5 1 e1000325 10.1371/journal.pgen.1000325 19119423 PMC2604467

[B4] Huntley S Baggott DM Hamilton AT Tran-Gyamfi M Yang S Kim J Gordon L Branscomb E Stubbs L A comprehensive catalog of human KRAB-associated zinc finger genes: insights into the evolutionary history of a large family of transcriptional repressors Genome Res 2006 16 5 669 677 10.1101/gr.4842106 16606702 PMC1457042

[B5] Tadepally HD Burger G Aubry M Evolution of C2H2-zinc finger genes and subfamilies in mammals: species-specific duplication and loss of clusters, genes and effector domains BMC Evol Biol 2008 8 176 10.1186/1471-2148-8-176 18559114 PMC2443715

[B6] Vaquerizas JM Kummerfeld SK Teichmann SA Luscombe NM A census of human transcription factors: function, expression and evolution Nat Rev Genet 2009 10 4 252 263 10.1038/nrg2538 19274049

[B7] Lorenz P Dietmann S Wilhelm T Koczan D Autran S Gad S Wen G Ding G Li Y Rousseau-Merck MF Thiesen HJ The ancient mammalian KRAB zinc finger gene cluster on human chromosome 8q24.3 illustrates principles of C2H2 zinc finger evolution associated with unique expression profiles in human tissues BMC Genomics 2010 11 206 10.1186/1471-2164-11-206 20346131 PMC2865497

[B8] Zheng L Pan H Li S Flesken-Nikitin A Chen PL Boyer TG Lee WH Sequence-specific transcriptional corepressor function for BRCA1 through a novel zinc finger protein, ZBRK1 Mol Cell 2000 6 4 757 768 10.1016/S1097-2765(00)00075-7 11090615

[B9] Li X Ito M Zhou F Youngson N Zuo X Leder P Ferguson-Smith AC A maternal-zygotic effect gene, Zfp57, maintains both maternal and paternal imprints Dev Cell 2008 15 4 547 557 10.1016/j.devcel.2008.08.014 18854139 PMC2593089

[B10] Tian C Xing G Xie P Lu K Nie J Wang J Li L Gao M Zhang L He F KRAB-type zinc-finger protein Apak specifically regulates p53-dependent apoptosis Nat Cell Biol 2009 11 5 580 591 10.1038/ncb1864 19377469

[B11] Margolin JF Friedman JR Meyer WK Vissing H Thiesen HJ Rauscher FJ Kruppel-associated boxes are potent transcriptional repression domains Proc Natl Acad Sci USA 1994 91 10 4509 4513 10.1073/pnas.91.10.4509 8183939 PMC43815

[B12] Friedman JR Fredericks WJ Jensen DE Speicher DW Huang XP Neilson EG Rauscher FJ KAP-1, a novel corepressor for the highly conserved KRAB repression domain Genes Dev 1996 10 16 2067 2078 10.1101/gad.10.16.2067 8769649

[B13] Kim SS Chen YM O'Leary E Witzgall R Vidal M Bonventre JV A novel member of the RING finger family, KRIP-1, associates with the KRAB-A transcriptional repressor domain of zinc finger proteins Proc Natl Acad Sci USA 1996 93 26 15299 15304 10.1073/pnas.93.26.15299 8986806 PMC26399

[B14] Moosmann P Georgiev O Le Douarin B Bourquin JP Schaffner W Transcriptional repression by RING finger protein TIF1 beta that interacts with the KRAB repressor domain of KOX1 Nucleic Acids Res 1996 24 24 4859 4867 10.1093/nar/24.24.4859 9016654 PMC146346

[B15] Le Douarin B Nielsen AL Garnier JM Ichinose H Jeanmougin F Losson R Chambon P A possible involvement of TIF1 alpha and TIF1 beta in the epigenetic control of transcription by nuclear receptors Embo J 1996 15 23 6701 6715 8978696 PMC452494

[B16] Ryan RF Schultz DC Ayyanathan K Singh PB Friedman JR Fredericks WJ Rauscher FJ KAP-1 corepressor protein interacts and colocalizes with heterochromatic and euchromatic HP1 proteins: a potential role for Kruppel-associated box-zinc finger proteins in heterochromatin-mediated gene silencing Mol Cell Biol 1999 19 6 4366 4378 10330177 10.1128/mcb.19.6.4366 PMC104396

[B17] Underhill C Qutob MS Yee SP Torchia J A novel nuclear receptor corepressor complex, N-CoR, contains components of the mammalian SWI/SNF complex and the corepressor KAP-1 J Biol Chem 2000 275 51 40463 40470 10.1074/jbc.M007864200 11013263

[B18] Peng H Begg GE Schultz DC Friedman JR Jensen DE Speicher DW Rauscher FJ Reconstitution of the KRAB-KAP-1 repressor complex: a model system for defining the molecular anatomy of RING-B box-coiled-coil domain-mediated protein-protein interactions J Mol Biol 2000 295 5 1139 1162 10.1006/jmbi.1999.3402 10653693

[B19] Schultz DC Friedman JR Rauscher FJ Targeting histone deacetylase complexes via KRAB-zinc finger proteins: the PHD and bromodomains of KAP-1 form a cooperative unit that recruits a novel isoform of the Mi-2alpha subunit of NuRD Genes Dev 2001 15 4 428 443 10.1101/gad.869501 11230151 PMC312636

[B20] Schultz DC Ayyanathan K Negorev D Maul GG Rauscher FJ SETDB1: a novel KAP-1-associated histone H3, lysine 9-specific methyltransferase that contributes to HP1-mediated silencing of euchromatic genes by KRAB zinc-finger proteins Genes Dev 2002 16 8 919 932 10.1101/gad.973302 11959841 PMC152359

[B21] Cammas F Herzog M Lerouge T Chambon P Losson R Association of the transcriptional corepressor TIF1beta with heterochromatin protein 1 (HP1): an essential role for progression through differentiation Genes Dev 2004 18 17 2147 2160 10.1101/gad.302904 15342492 PMC515292

[B22] Wiznerowicz M Jakobsson J Szulc J Liao S Quazzola A Beermann F Aebischer P Trono D The Kruppel-associated box repressor domain can trigger de novo promoter methylation during mouse early embryogenesis J Biol Chem 2007 282 47 34535 34541 10.1074/jbc.M705898200 17893143

[B23] Ayyanathan K Lechner MS Bell P Maul GG Schultz DC Yamada Y Tanaka K Torigoe K Rauscher FJ Regulated recruitment of HP1 to a euchromatic gene induces mitotically heritable, epigenetic gene silencing: a mammalian cell culture model of gene variegation Genes Dev 2003 17 15 1855 1869 10.1101/gad.1102803 12869583 PMC196232

[B24] Sripathy SP Stevens J Schultz DC The KAP1 corepressor functions to coordinate the assembly of de novo HP1-demarcated microenvironments of heterochromatin required for KRAB zinc finger protein-mediated transcriptional repression Mol Cell Biol 2006 26 22 8623 8638 10.1128/MCB.00487-06 16954381 PMC1636786

[B25] O'Geen H Squazzo SL Iyengar S Blahnik K Rinn JL Chang HY Green R Farnham PJ Genome-wide analysis of KAP1 binding suggests autoregulation of KRAB-ZNFs PLoS Genet 2007 3 6 e89 10.1371/journal.pgen.0030089 17542650 PMC1885280

[B26] Iyengar S Ivanov AV Jin VX Rauscher FJ Farnham PJ Functional analysis of KAP1 genomic recruitment Mol Cell Biol 2011 10.1128/MCB.01331-10 PMC3133220 21343339

[B27] Loyola A Tagami H Bonaldi T Roche D Quivy JP Imhof A Nakatani Y Dent SY Almouzni G The HP1alpha-CAF1-SetDB1-containing complex provides H3K9me1 for Suv39-mediated K9me3 in pericentric heterochromatin EMBO Rep 2009 10 7 769 775 10.1038/embor.2009.90 19498464 PMC2727428

[B28] Groner AC Meylan S Ciuffi A Zangger N Ambrosini G Denervaud N Bucher P Trono D KRAB-zinc finger proteins and KAP1 can mediate long-range transcriptional repression through heterochromatin spreading PLoS Genet 2010 6 3 e1000869 10.1371/journal.pgen.1000869 20221260 PMC2832679

[B29] Deuschle U Meyer WK Thiesen HJ Tetracycline-reversible silencing of eukaryotic promoters Mol Cell Biol 1995 15 4 1907 1914 7891684 10.1128/mcb.15.4.1907 PMC230416

[B30] Moosmann P Georgiev O Thiesen HJ Hagmann M Schaffner W Silencing of RNA polymerases II and III-dependent transcription by the KRAB protein domain of KOX1, a Kruppel-type zinc finger factor Biol Chem 1997 378 7 669 677 10.1515/bchm.1997.378.7.669 9278146

[B31] Schroder AR Shinn P Chen H Berry C Ecker JR Bushman F HIV-1 integration in the human genome favors active genes and local hotspots Cell 2002 110 4 521 529 10.1016/S0092-8674(02)00864-4 12202041

[B32] Mitchell RS Beitzel BF Schroder AR Shinn P Chen H Berry CC Ecker JR Bushman FD Retroviral DNA integration: ASLV, HIV, and MLV show distinct target site preferences PLoS Biol 2004 2 8 E234 10.1371/journal.pbio.0020234 15314653 PMC509299

[B33] Margulies M Egholm M Altman WE Attiya S Bader JS Bemben LA Berka J Braverman MS Chen YJ Chen Z Dewell SB Du L Fierro JM Gomes XV Godwin BC He W Helgesen S Ho CH Irzyk GP Jando SC Alenquer ML Jarvie TP Jirage KB Kim JB Knight JR Lanza JR Leamon JH Lefkowitz SM Lei M Li J Lohman KL Lu H Makhijani VB McDade KE McKenna MP Myers EW Nickerson E Nobile JR Plant R Puc BP Ronan MT Roth GT Sarkis GJ Simons JF Simpson JW Srinivasan M Tartaro KR Tomasz A Vogt KA Volkmer GA Wang SH Wang Y Weiner MP Yu P Begley RF Rothberg JM Genome sequencing in microfabricated high-density picolitre reactors Nature 2005 437 7057 376 380 16056220 10.1038/nature03959 PMC1464427

[B34] Wang GP Ciuffi A Leipzig J Berry CC Bushman FD HIV integration site selection: analysis by massively parallel pyrosequencing reveals association with epigenetic modifications Genome Res 2007 17 8 1186 1194 10.1101/gr.6286907 17545577 PMC1933515

[B35] Iseli C Ambrosini G Bucher P Jongeneel CV Indexing strategies for rapid searches of short words in genome sequences PLoS One 2007 2 6 e579 10.1371/journal.pone.0000579 17593978 PMC1894650

[B36] Lewinski MK Yamashita M Emerman M Ciuffi A Marshall H Crawford G Collins F Shinn P Leipzig J Hannenhalli S Berry CC Ecker JR Bushman FD Retroviral DNA integration: viral and cellular determinants of target-site selection PLoS Pathog 2006 2 6 e60 10.1371/journal.ppat.0020060 16789841 PMC1480600

[B37] De Palma M Montini E Santoni de Sio FR Benedicenti F Gentile A Medico E Naldini L Promoter trapping reveals significant differences in integration site selection between MLV and HIV vectors in primary hematopoietic cells Blood 2005 105 6 2307 2315 10.1182/blood-2004-03-0798 15542582

[B38] Berry C Hannenhalli S Leipzig J Bushman FD Selection of target sites for mobile DNA integration in the human genome PLoS Comput Biol 2006 2 11 e157 10.1371/journal.pcbi.0020157 17166054 PMC1664696

[B39] Cuddapah S Jothi R Schones DE Roh TY Cui K Zhao K Global analysis of the insulator binding protein CTCF in chromatin barrier regions reveals demarcation of active and repressive domains Genome Res 2009 19 1 24 32 19056695 10.1101/gr.082800.108 PMC2612964

[B40] Jin C Zang C Wei G Cui K Peng W Zhao K Felsenfeld G H3.3/H2A.Z double variant-containing nucleosomes mark 'nucleosome-free regions' of active promoters and other regulatory regions Nat Genet 2009 41 8 941 945 10.1038/ng.409 19633671 PMC3125718

[B41] Wang Z Zang C Cui K Schones DE Barski A Peng W Zhao K Genome-wide mapping of HATs and HDACs reveals distinct functions in active and inactive genes Cell 2009 138 5 1019 1031 10.1016/j.cell.2009.06.049 19698979 PMC2750862

[B42] West AG Gaszner M Felsenfeld G Insulators: many functions, many mechanisms Genes Dev 2002 16 3 271 288 10.1101/gad.954702 11825869

[B43] Gaszner M Felsenfeld G Insulators: exploiting transcriptional and epigenetic mechanisms Nat Rev Genet 2006 7 9 703 713 10.1038/nrg1925 16909129

[B44] Recillas-Targa F Pikaart MJ Burgess-Beusse B Bell AC Litt MD West AG Gaszner M Felsenfeld G Position-effect protection and enhancer blocking by the chicken beta-globin insulator are separable activities Proc Natl Acad Sci USA 2002 99 10 6883 6888 10.1073/pnas.102179399 12011446 PMC124498

[B45] Hediger F Gasser SM Heterochromatin protein 1: don't judge the book by its cover! Curr Opin Genet Dev 2006 16 2 143 150 10.1016/j.gde.2006.02.013 16503133

[B46] Blahnik KR Dou L Echipare L Iyengar S O'Geen H Sanchez E Zhao Y Marra MA Hirst M Costello JF Korf I Farnham PJ Characterization of the contradictory chromatin signatures at the 3' exons of zinc finger genes PLoS One 2011 6 2 e17121 10.1371/journal.pone.0017121 21347206 PMC3039671

[B47] Frietze S O'Geen H Blahnik KR Jin VX Farnham PJ ZNF274 recruits the histone methyltransferase SETDB1 to the 3' ends of ZNF genes PLoS One 2010 5 12 e15082 10.1371/journal.pone.0015082 21170338 PMC2999557

[B48] Loenarz C Ge W Coleman ML Rose NR Cooper CD Klose RJ Ratcliffe PJ Schofield CJ PHF8, a gene associated with cleft lip/palate and mental retardation, encodes for an Nepsilon-dimethyl lysine demethylase Hum Mol Genet 2010 19 2 217 222 10.1093/hmg/ddp480 19843542 PMC4673897

[B49] Kleine-Kohlbrecher D Christensen J Vandamme J Abarrategui I Bak M Tommerup N Shi X Gozani O Rappsilber J Salcini AE Helin K A functional link between the histone demethylase PHF8 and the transcription factor ZNF711 in X-linked mental retardation Mol Cell 2010 38 2 165 178 10.1016/j.molcel.2010.03.002 20346720 PMC2989439

[B50] Mackay DJ Callaway JL Marks SM White HE Acerini CL Boonen SE Dayanikli P Firth HV Goodship JA Haemers AP Hahnemann JM Kordonouri O Masoud AF Oestergaard E Storr J Ellard S Hattersley AT Robinson DO Temple IK Hypomethylation of multiple imprinted loci in individuals with transient neonatal diabetes is associated with mutations in ZFP57 Nat Genet 2008 40 8 949 951 10.1038/ng.187 18622393

[B51] Henikoff S Position-effect variegation after 60 years Trends Genet 1990 6 12 422 426 2087785 10.1016/0168-9525(90)90304-o

[B52] Wiznerowicz M Trono D Conditional suppression of cellular genes: lentivirus vector-mediated drug-inducible RNA interference J Virol 2003 77 16 8957 8961 10.1128/JVI.77.16.8957-8951.2003 12885912 PMC167245

[B53] Sheehy AM Gaddis NC Choi JD Malim MH Isolation of a human gene that inhibits HIV-1 infection and is suppressed by the viral Vif protein Nature 2002 418 6898 646 650 10.1038/nature00939 12167863

[B54] Hsu JY Sun ZW Li X Reuben M Tatchell K Bishop DK Grushcow JM Brame CJ Caldwell JA Hunt DF Lin R Smith MM Allis CD Mitotic phosphorylation of histone H3 is governed by Ipl1/aurora kinase and Glc7/PP1 phosphatase in budding yeast and nematodes Cell 2000 102 3 279 291 10.1016/S0092-8674(00)00034-9 10975519

[B55] DeLong ER DeLong DM Clarke-Pearson DL Comparing the areas under two or more correlated receiver operating characteristic curves: a nonparametric approach Biometrics 1988 44 3 837 845 10.2307/2531595 3203132

[B56] Carson JP Zhang N Frampton GM Gerry NP Lenburg ME Christman MF Pharmacogenomic identification of targets for adjuvant therapy with the topoisomerase poison camptothecin Cancer Res 2004 64 6 2096 2104 10.1158/0008-5472.CAN-03-2029 15026349

[B57] Cassani B Montini E Maruggi G Ambrosi A Mirolo M Selleri S Biral E Frugnoli I Hernandez-Trujillo V Di Serio C Roncarolo MG Naldini L Mavilio F Aiuti A Integration of retroviral vectors induces minor changes in the transcriptional activity of T cells from ADA-SCID patients treated with gene therapy Blood 2009 114 17 3546 3556 10.1182/blood-2009-02-202085 19652199

[B58] Irizarry RA Hobbs B Collin F Beazer-Barclay YD Antonellis KJ Scherf U Speed TP Exploration, normalization, and summaries of high density oligonucleotide array probe level data Biostatistics 2003 4 2 249 264 10.1093/biostatistics/4.2.249 12925520

