# Supplementary figures and images for: A gene-rich, transcriptionally active environment and the pre-deposition of repressive marks are predictive of susceptibility to KRAB/KAP1-mediated silencing
Source: BMC Genomics. 2011 Jul 26;12:378. doi: 10.1186/1471-2164-12-378 (PMC3199781; doi:10.1186/1471-2164-12-378)

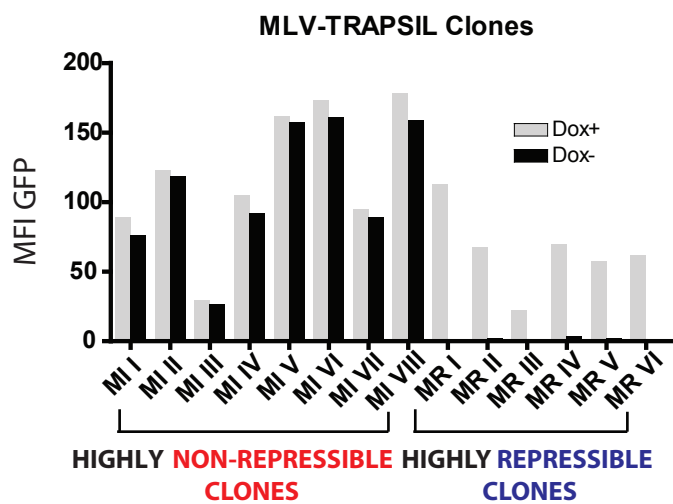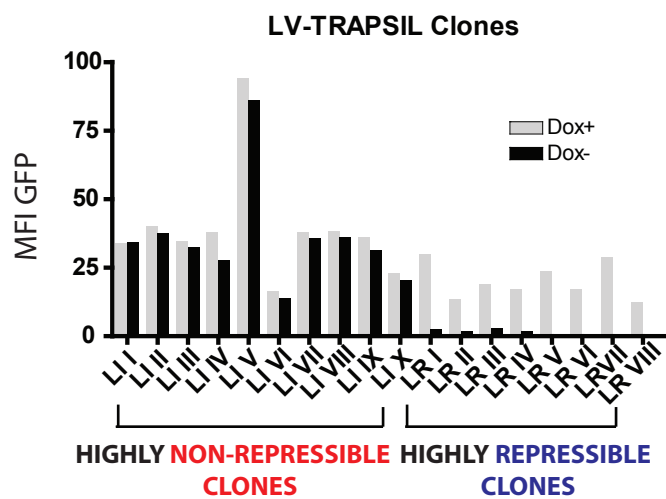

Supplement: Additional file 2 — KRAB/KAP1 repression profiles from clones isolated from repressible and non-repressible TrapSil populations. Reporter gene activity in the presence and absence of doxycycline (Dox) was monitored in clones derived from repressible and non-repressible TrapSil populations. [file 1471-2164-12-378-S2.PDF]

**A**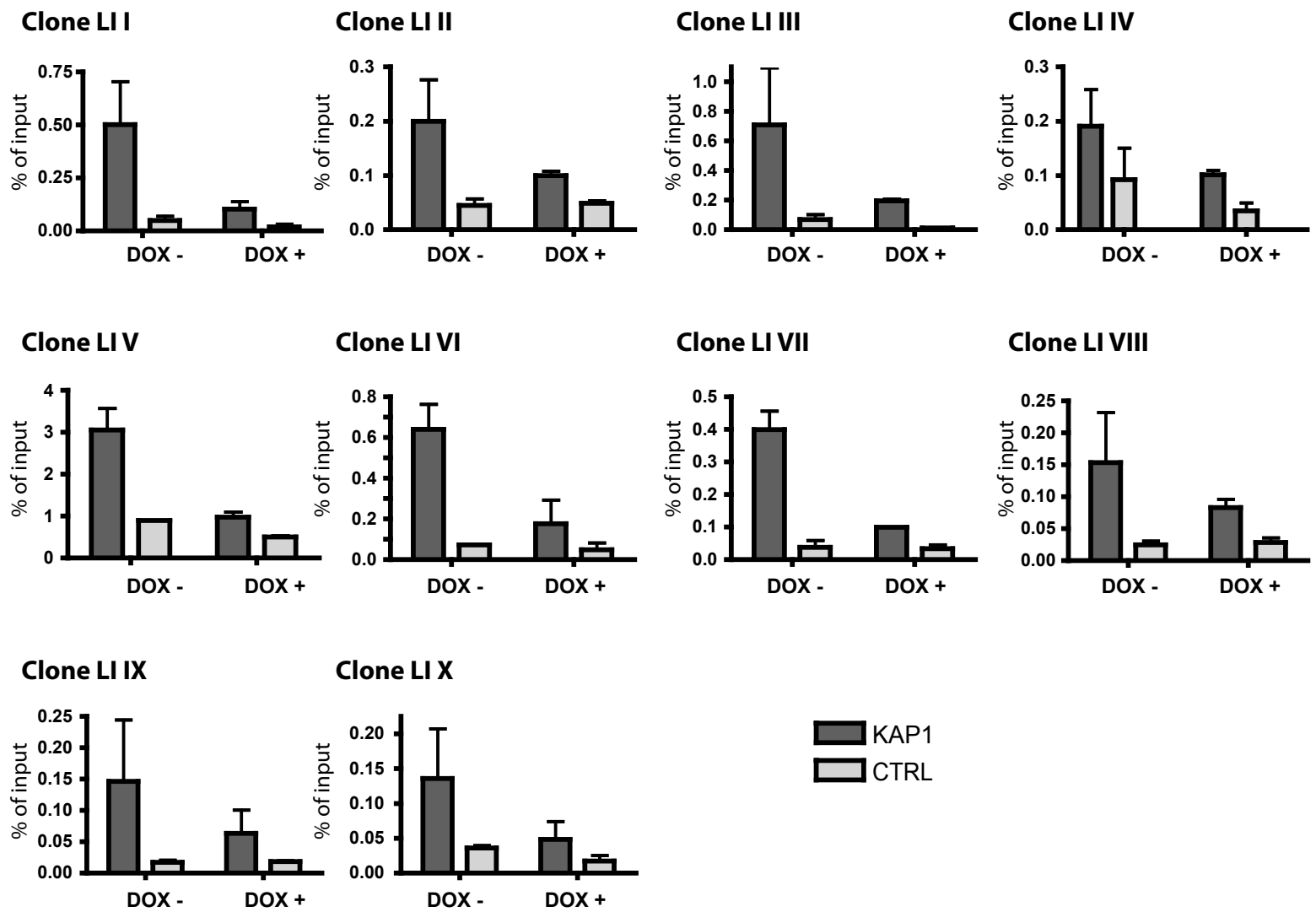**B**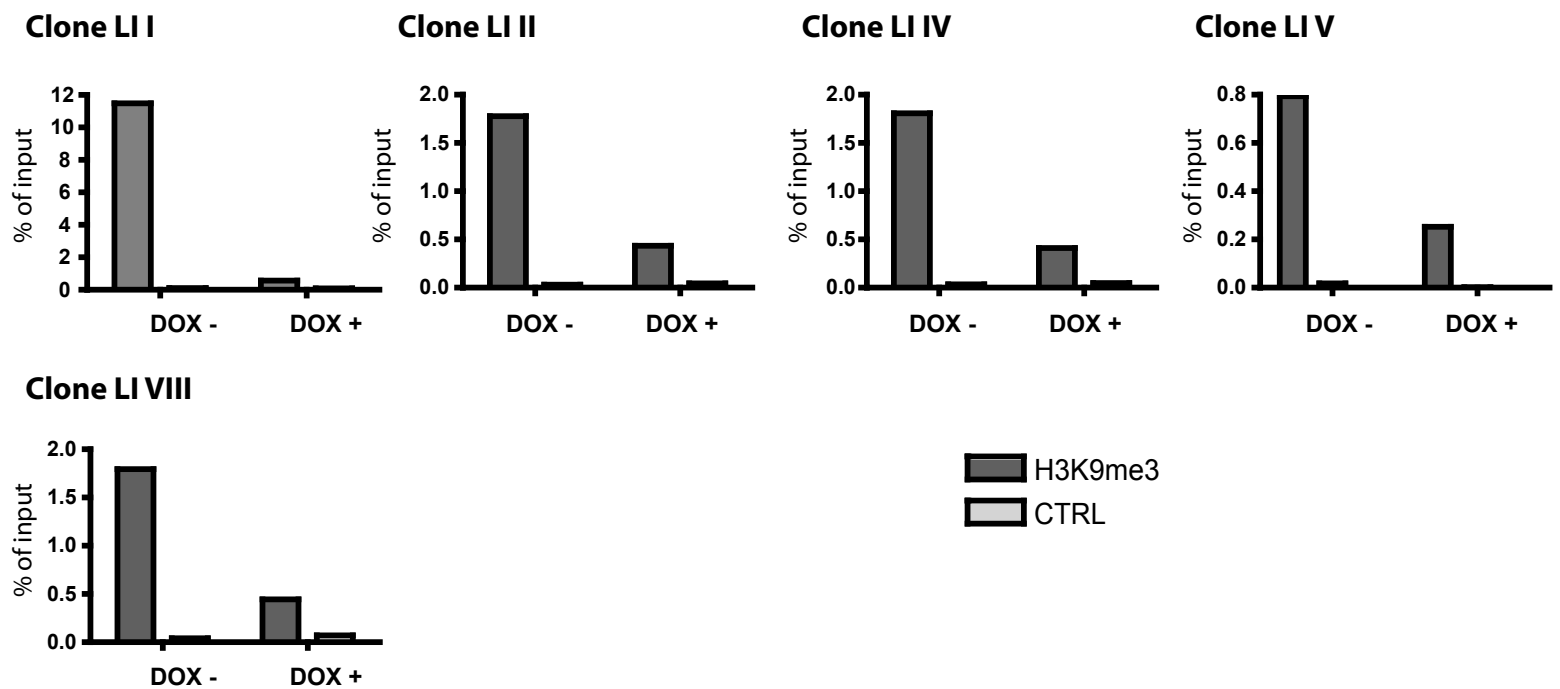

Supplement: Additional file 3 — KAP1 and H3K9me3 are present at non-repressible clones in the presence of repressor binding. Chromatin-immunoprecipitation (ChIP) in combination with qPCR was used to verify proper KAP1 recruitment to gene traps in the absence of doxycycline in non-repressible clones. In addition, levels of H3K9me3 monitored the enzymatic activity of the KRAB/KAP1 silencing complex. The relative enrichment was calculated as a percentage of the total input (% of total input). [file 1471-2164-12-378-S3.PDF]

**A**

**GROUP 1                      GROUP 2                      GROUP 3**

**H3.3**

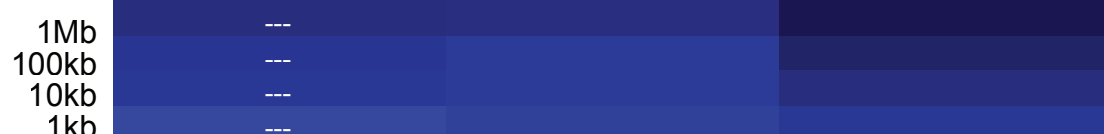

**H2Az**

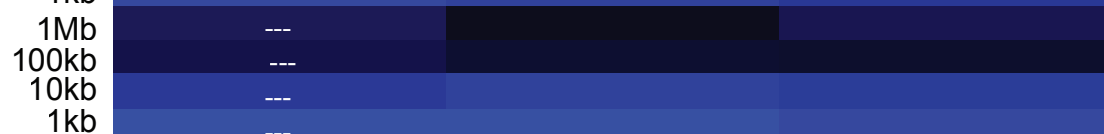

**B**

**CTCF**

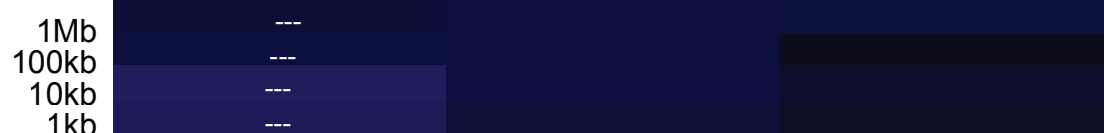

**C**

**P300**

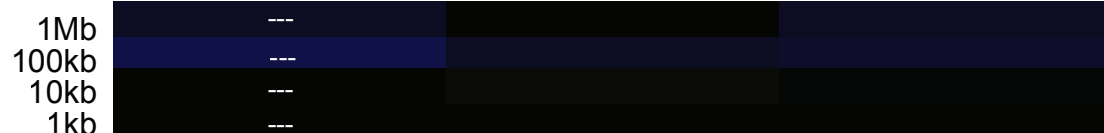

**CD4-TIP60**

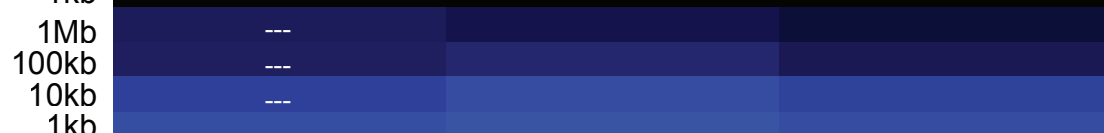

**CD4-PCAF**

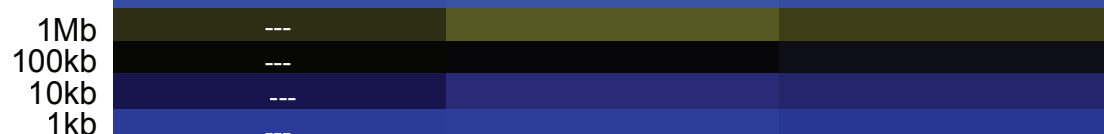

**CD4-MOF**

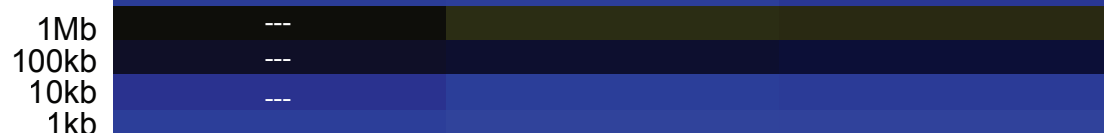

**CD4-HDAC6**

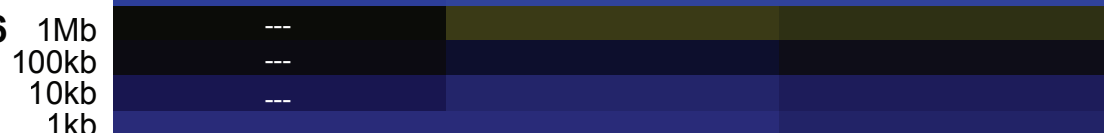

**CD4-HDAC3**

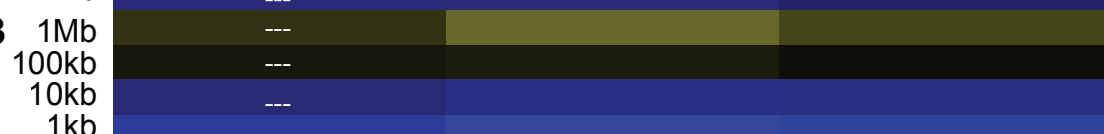

**CD4-HDAC2**

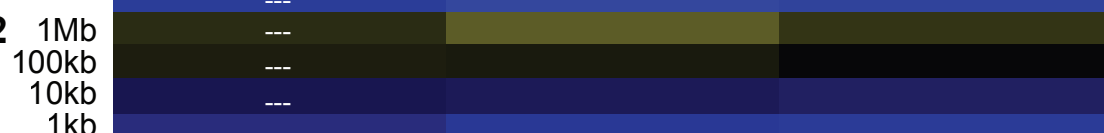

**CD4-HDAC1**

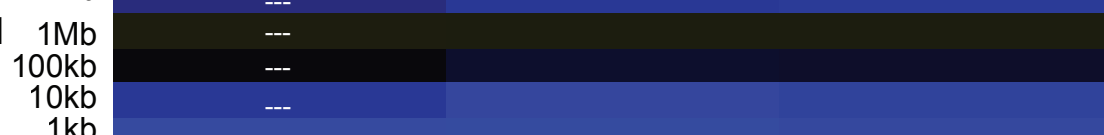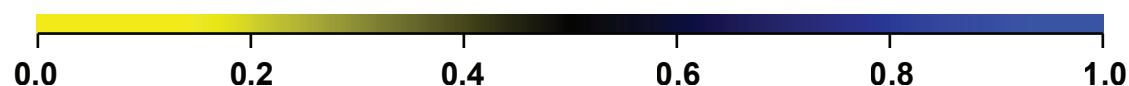

Supplement: Additional file 5 — Heterochromatin barrier elements in the environment of the matched gene groups. The relative level of barrier elements was assayed in the three gene groups (group 1: "long-range repression", group 2: "limited repression" and group 3: "absence of repression"). Calculations were based on ROC curves as described in Figure 3 and included various DNA stretches around the promoters of the different gene groups. Non-central chi square statistical analysis indicated no differences between the groups. The features considered in the analysis were (A) Histone variants associated with active remodeling (H3.3, H2Az), (B) CTCF or (C) chromatin modifiers, such as the histone acetyltransferases p300, TIP60, PCAF and MOF, in addition to the histone deacetylases HDAC1, 2, 3, and 6. The genome-wide binding data for these factors came from published work in HeLa cells (H3.3, H2Az, p300) or CD4 cells (TIP60, PCAF, MOF, HDAC1, 2, 3 and 6). [file 1471-2164-12-378-S5.PDF]

A

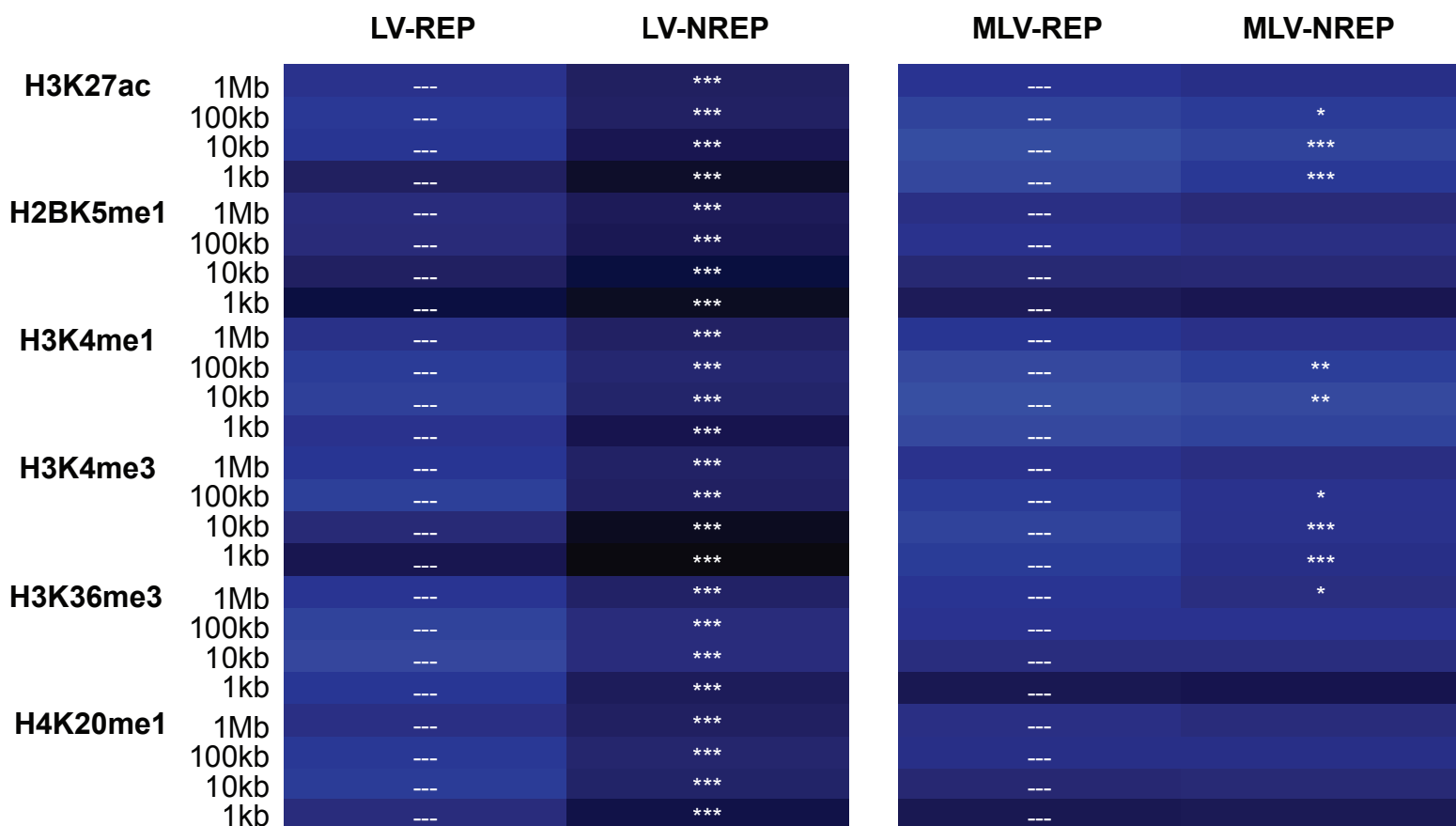

B

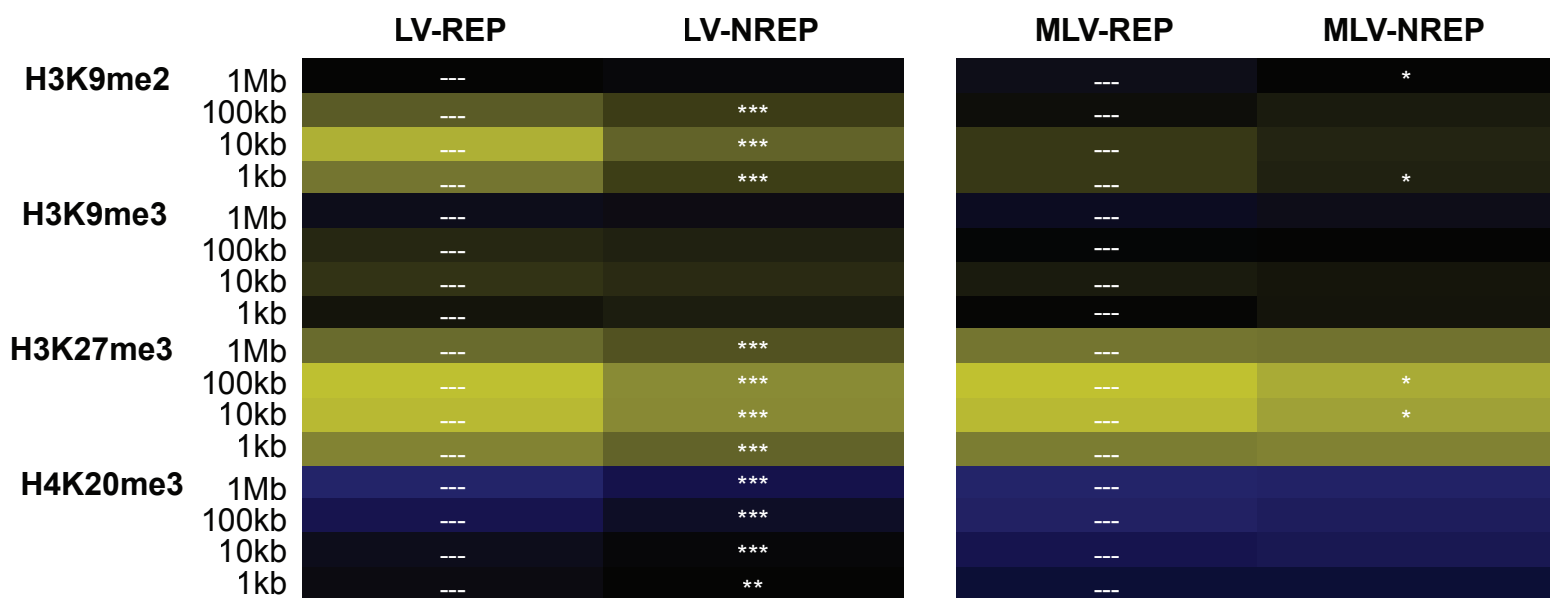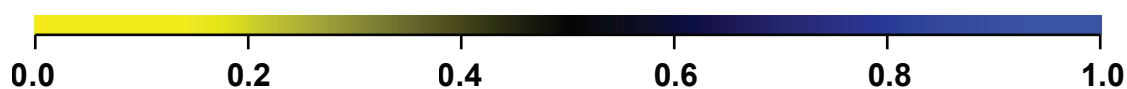

Supplement: Additional file 6 — Characterization of the chromatin environment of all KRAB/KAP1-docking integrants. LV and MLV TrapSil integrants were split in repressing (REP) and non-repressing (NREP) groups according to the effect of KRAB/KAP1 recruitment on the trapped promoters. The chromatin environment of the different proviral integrant groups was analyzed for the indicated features by ROC curve analysis. (A) The histone modifications in the analysis included H3K27ac, H2BK5me1, H3K4me1, H3K4me3, H3K36me3, H4K20me1, which are mostly found within active chromatin. (B) The histone modifications in this analysis included H3K9me2, H3K9me3, H3K27me3 and H4K20me3, mainly associated with silent chromatin. Non-central chi-square statistical analysis compared differences between the integrant groups with the differential silencing phenotypes. P-Value Legend: * p < 0.05; ** p < 0.01; *** p < 0.001. [file 1471-2164-12-378-S6.PDF]

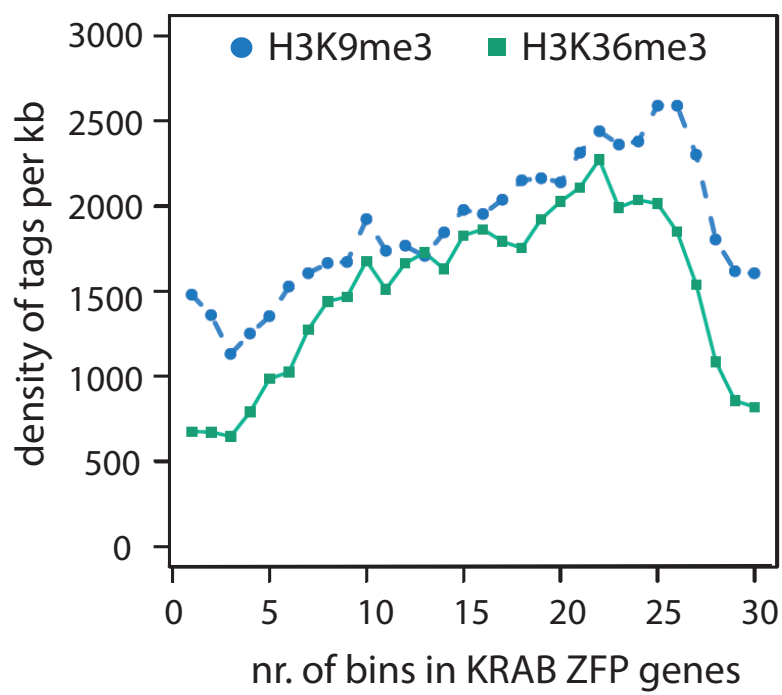

Supplement: Additional file 7 — Characterization of the chromatin state of KRAB-ZFP genes. Illustration of H3K9me3 and H3K36me3 enrichments at KRAB-ZFP gene bodies in HeLa cells. KRAB-ZFP gene lengths were equalized by division into 40 bins and included a 2 kb flanking region on both sides of the genes. See Additional File 8 for the list of KRAB-ZFP genes, which were included in the analysis. [file 1471-2164-12-378-S7.PDF]

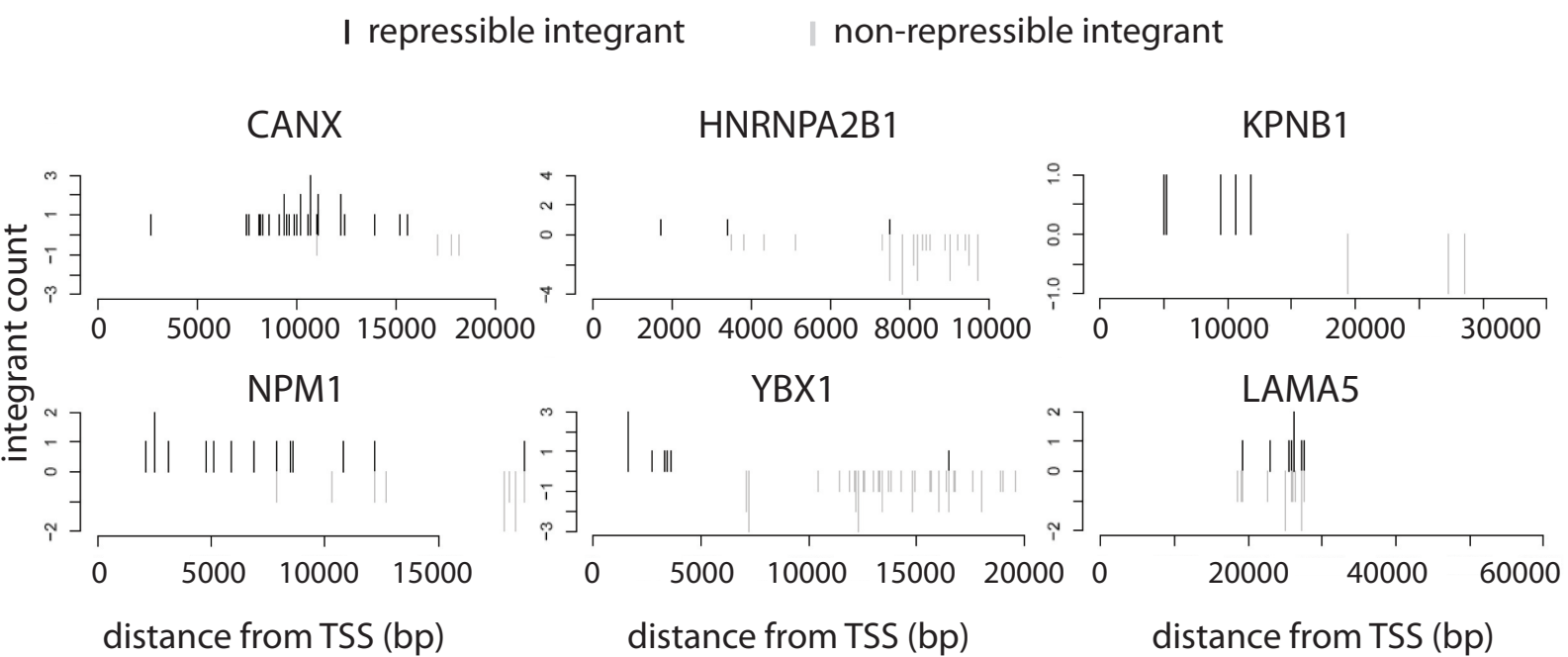

Supplement: Additional file 9 — Genes with multiple TrapSil integrants of different silencing phenotypes. Graphic representation of 6 genes, where multiple TrapSil integrations of different phenotypes have occurred: calnexin precursor (CANX), heterogeneous nuclear ribonucleoprotein A2/B1 (HNRNPA2B1), karyopherin beta 1 (KPNB1), nucleophosmin 1 (NPM1), Y-box binding protein 1 (YBX1), laminin alpha 5 (LAMA5). The integrants with a repressible phenotype are depicted as black lines above the baseline (y = 0), whereas the non-repressible counterparts are depicted as light grey lines below the baseline. Some intragenic sites carried multiple but distinct proviral integrants and subsequently harboured more than 1 integrant count (see y-axis). [file 1471-2164-12-378-S9.PDF]
